# Nitrogen-Doped Porous Carbons Derived from Peanut Shells as Efficient Electrodes for High-Performance Supercapacitors

**DOI:** 10.3390/ijms25147583

**Published:** 2024-07-10

**Authors:** Shibo Liu, Qishan Zhang, Jiani Liu, Jiarui Li, Wenjia Liu, Yuan Wang, Shaojun Yuan

**Affiliations:** Low-Carbon Technology & Chemical Reaction Engineering Lab, College of Chemical Engineering, Sichuan University, Chengdu 610065, China; liushibo@stu.scu.edu.cn (S.L.); zhangqishan@stu.scu.edu.cn (Q.Z.); liujiani@stu.scu.edu.cn (J.L.); 2022141490313@stu.scu.edu.cn (J.L.); 19980811423@163.com (W.L.)

**Keywords:** biomass, peanut shells, supercapacitors, nitrogen doping, porous activated carbon

## Abstract

The doping of porous carbon materials with nitrogen is an effective approach to enhance the electrochemical performance of electrode materials. In this study, nitrogen-doped porous carbon derived from peanut shells was prepared as an electrode for supercapacitors. Melamine, urea, urea phosphate, and ammonium dihydrogen phosphate were employed as different nitrogen dopants. The optimized electrode material PA-1-1 prepared by peanut shells, with ammonium dihydrogen phosphate as a nitrogen dopant, exhibited a N content of 3.11% and a specific surface area of 602.7 m^2^/g. In 6 M KOH, the PA-1-1 electrode delivered a high specific capacitance of 208.3 F/g at a current density of 1 A/g. Furthermore, the PA-1-1 electrode demonstrated an excellent rate performance with a specific capacitance of 170.0 F/g (retention rate of 81.6%) maintained at 20 A/g. It delivered a capacitance of PA-1-1 with a specific capacitance retention of 98.8% at 20 A/g after 5000 cycles, indicating excellent cycling stability. The PA-1-1//PA-1-1 symmetric supercapacitor exhibited an energy density of 17.7 Wh/kg at a power density of 2467.0 W/kg. This work not only presents attractive N-doped porous carbon materials for supercapacitors but also offers a novel insight into the rational design of biochar carbon derived from waste peelings.

## 1. Introduction

In the context of energy shortage and ecosystem destruction, there is an increasing interest in high-power and high-energy density storage systems [1,2]. Supercapacitors have become promising candidates for reversible, efficient, and convenient power storage and release [3,4]. Among all the various electrochemical energy storage devices, supercapacitors have attracted more attention for their advantages of ultra-high capacitors, high power density, super-long cycle life, and high charge and discharge efficiency [5,6]. Electric double-layer capacitors (EDLCs) play a critical role in satisfying the demand for electronic devices and systems for their long lifetime, fast charging speed, and environmentally friendly nature [7,8]. While the energy density of EDLCs is limited, there is still a gap with traditional lithium batteries owing to no electrochemical reactions being involved at the electrolyte and electrode interface [9]. Consequently, it is imperative to enhance the electrode material to optimize the faradaic reaction, thereby promoting the specific capacitance.

Carbon materials are promising electrode materials due to a wide range of sources, low cost, and good chemical stability in many solutions [10,11]. The main supercapacitor materials studied more are activated carbon, activated carbon fiber, carbon aerogel, carbon nanotubes, etc., among which activated carbons have a long history of industrial production and application [12,13]. Their rich pore structure and huge specific surface area give them strong adsorption and catalytic properties, which make them widely used in many fields [14]. There are abundant sources of raw materials that can be used to make activated carbon, and generally, materials that are rich in carbon can be used to prepare activated carbon. Recently, various natural biomass resources have been widely used to prepare carbon-based electrode materials for electrochemical energy storage devices due to their advantages of low cost, renewability, and environmental friendliness [15,16,17].

Biomass is an environmentally friendly, renewable, and abundant type of carbon resource, which has a natural biological structure suitable for the formation of porous nanostructures [18,19]. Therefore, it is considered a promising precursor for activated carbon production. Peanuts, as a kind of biomass, are one of the most widely grown oilseed crops in the world, and peanut shells are one of the main by-products of the production process [20,21]. Zhan et al. [22] synthesized three-dimensional porous biomass charcoal from peanut shells using KOH as an activator and the results show that the gravimetric specific capacitance of KOH-AC reached 440 F/g at 0.1 A/g and retained 286 F/g at 10 A/g. Yadav et al. [23] performed two different pre-treatments (ethanol soaking and hydrothermal) on peanut shells and the results were the AC electrodes offer a superior performance in terms of specific capacitance (189 F/g). The EDLC showed a stable performance up to 10,000 charge/discharge cycles with a 28% initial fading in specific capacitance. Guo et al. [24] developed a one-step bimetallic activation method to prepare peanut shell biomass-activated carbon for supercapacitors by the activation properties of metal activators and the etching effect of CO_2_. The results indicate that the FeCl_3_/MgCl_2_-activated sample at 800 °C exhibited the maximum discharge specific capacitance of 247.2 F/g at a current density of 1 A/g in the 1 M Na_2_SO_4_ electrolyte and a good rate capability of 202.3 F/g at 10 A/g. Furthermore, a high cyclic stability was also obtained with a capacitance retention of 96.31% after 5000 cycles. Qiao et al. [25] prepared activated biochar materials from peanut shells by pyrolysis and heat treatment with molten KCl, and the results showed an outstanding super capacitance performance in 6 M KOH with a high specific capacitance (355 F/g at 0.5 A/g), which exceeds all reported biochar derived from peanut shells.

The electrochemical performance of biomass-activated carbons as electrode materials for supercapacitors depends mainly on their specific surface area, pore size distribution, and functional groups [26]. Therefore, to obtain highly available biomass-derived carbons in supercapacitor applications, it is necessary to develop porous carbons with rich functional groups and more reasonable pore structures. In general, it is believed that doping the activated carbon skeleton with heteroatoms can effectively modulate the electronic properties of carbon materials and enhance the hydrophilicity and electrical conductivity of carbon materials [27]. In addition, heteroatoms can create pseudo-capacitance by reacting with functional groups on the surface of the carbon material, thereby improving the electrical conductivity and wettability of the carbon material, and the overall electrochemical properties can be improved [28]. Among the various non-metallic elements that can be doped into carbon materials, those containing N are particularly useful. This is because the radii of N and C atoms are almost identical, which allows the N atoms to be incorporated into the carbon structure with ease [29]. N-type doping sites, which exhibit electron-donating behaviors, are typically created by N atoms. These sites play a crucial role in enhancing the electronic conductivity by increasing the electron density within the π-π conjugation of the carbon matrix, optimizing the surface wettability, facilitating charge transfer by charge modulation, and ultimately leading to an increase in the power and energy density [30]. The capacitance of nitrogen-doped carbons (NPCs) can be attributed to two sources: the EDLC effect and the faradaic reaction occurring on or close to the surface of the carbon material [31]. Song et al. [32] prepared a corncob-based porous carbon by KOH activation and carbonization, followed by N doping employing urea. The specific capacitance of the optimized sample reached 382.6 F/g at 0.5 A/g and still maintained 232.7 F/g at a high current density of 20 A/g. Wang et al. [33] used grapes, KOH, and NH_4_Cl as biomass sources, activators, and nitrogen sources. The optimized sample NGHPC exhibited a unique honeycomb-like structure and a high specific surface area of 1268 m^2^/g. As a supercapacitor electrode, the NGPHC electrode exhibited a remarkable specific capacitance of 275 F/g at 0.5 A/g in a three-electrode cell. Zhang et al. [34] obtained nitrogen-doped porous carbon materials by the one-pot co-pyrolysis of a carbon precursor (wheat straw), nitrogen precursor (melamine), and salt templating. The optimized sample with 7.78% nitrogen content exhibited an excellent gravimetric capacitance of 223.9 F/g and maintained 91.4% capacitance after 10,000 cycles. In general, the preparation of NPCs is typically achieved through the pyrolysis of biomass waste containing nitrogen elements, followed by the post-treatment of carbon materials with nitrogen dopants at high temperatures [35]. Therefore, we hypothesized that the pyrolysis of peanut shells with different nitrogen resources would result in different active sites for carbon-based electrodes, which could further influence the electrochemical performance of SCs.

Accordingly, this study aims to synthesize porous and nitrogen-doped activated carbons from low-cost, abundant, and renewable biomass sources. Peanut shells were used as the carbon precursor. Melamine, urea, urea phosphate, and ammonium dihydrogen phosphate were employed as various nitrogen dopants, respectively. In addition, the amount of activator introduced was also investigated to determine the effect of the activator on the porosity of the biochar. A synthesis method that combines hydrothermal carbonization with KOH/N_2_ activation was devised. The optimized electrode material PA-1-1 (the mass ratio of peanut shell hydrocode to peanut shell is 1:1, and the mass ratio of KOH to peanut shell hydrocode is 1:1) prepared by peanut shells exhibited a N content of 3.11% and a specific surface area of 602.7 m^2^/g. The PA-1-1 electrode delivered a capacitance of 208.3 and 170 F/g at a current density of 1 and 20 A/g in 6 M KOH, respectively. Furthermore, it delivered a capacitance of PA-1-1 with a specific capacitance retention of 98.8% at 20 A/g after 5000 cycles, showing excellent cycling stability.

## 2. Results and Discussion

### 2.1. Characterization of Materials

**Figure 1:**
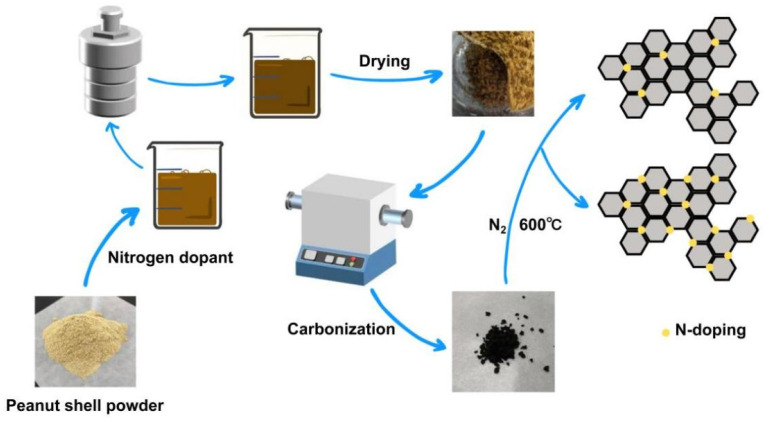
Schematic of the synthesis process for the PX-1-1 samples.

Figure 1 shows the schematic of the synthesis process for the PX-1-1 samples. The peanut shell powder and different nitrogen dopants were first mixed as the carbon and the nitrogen resource, respectively. Subsequently, the mixture was further pyrolyzed under a N_2_ atmosphere with KOH as the activator. Finally, the nitrogen-doped porous carbon was collected after washing with HCl to remove impurities.

**Figure 2:**
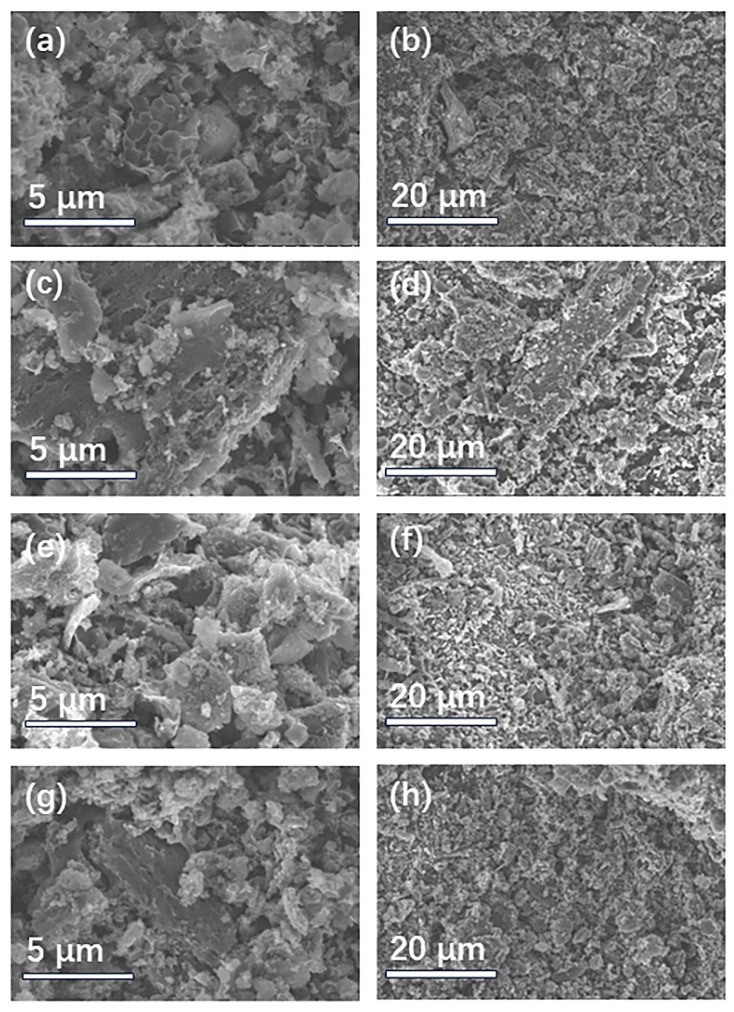
SEM of peanut shell-based materials PX-1-1: (**a**,**b**) PM-1-1, (**c**,**d**) PU-1-1, (**e**,**f**) PA-1-1, (**g**,**h**) PP-1-1.

Figure 2 depicts the scanning electron microscopy (SEM) images of the PX-1-1 samples doped with four nitrogen dopants at different magnifications. It can be observed that after nitrogen doping and potassium hydroxide (KOH) activation, the surface of all the PX-1-1 samples becomes very rough and presents very small fragments [36]. The EDS mapping images (Figure S1) show that the N and P elements are distributed on PA-1-1. However, all the samples exhibited irregular particle morphology under low-magnification SEM. It can be observed that, despite the differing types of nitrogen dopants, PM-1-1 (Figure 2a,b), PU-1-1 (Figure 2c,d), PA-1-1 (Figure 2e,f), and PP-1-1 (Figure 2g,h) exhibited a disordered carbon structure at 600 °C, indicating that the nitrogen source had no discernible effect on the morphology.

Furthermore, the influence of the mass ratio of KOH to peanut shell hydrocode on the morphology of the activated carbon was also investigated. The prepared materials were named PA-1-Y, where Y represents the mass ratio of KOH to peanut shell hydrocode (0:1, 1:1, 1:2, and 1:3). As illustrated in Figure S2, with the increase in the KOH ratio, all the carbon materials exhibited amorphous particle morphology, indicating that there is no direct correlation between the increase in KOH and the morphological characteristics observed in the SEM at 600 °C.

**Figure 3:**
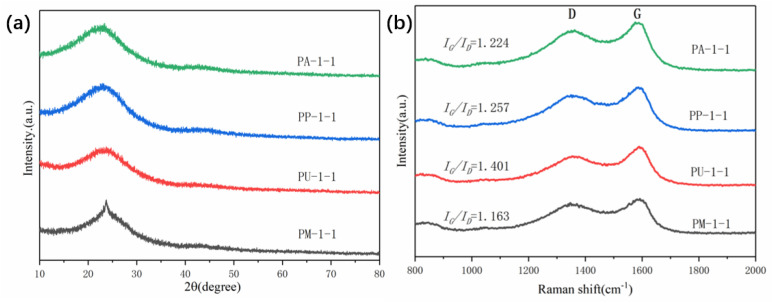
(**a**) XRD patterns of PX-1-1 (X = A, P, U, and M), and (**b**) Raman spectra of PX-1-1.

Figure 3a illustrates the XRD spectra of the PX-1-1 materials with varying types of nitrogen dopants at a fixed KOH dosage of KOH: PX = 1:1 (mass ratio). It can be observed that all the samples exhibit significant broad diffraction peaks in the range of 20–30°, indicating that the prepared samples have disordered carbon structures. Furthermore, no additional impurity peaks were observed in the graph, indicating that impurities can be effectively removed through acid washing. The lattice defects and morphology of the material were analyzed using Raman characterization testing. As illustrated in Figure 3b, all the samples exhibit two pronounced peaks at approximately 1350 cm^−1^ and 1590 cm^−1^, corresponding to the D and G bands, respectively. The D peak represents a defect in the C atom crystal and the G peak represents an in-plane stretching vibration of the sp^2^ hybridization of the C atom. Consequently, the magnitude of the I_G_/I_D_ values can be employed to facilitate the comparison of the degree of defects in different materials [37]. The I_G_/I_D_ values also exhibit different values when different nitrogen dopants are employed, indicating that the introduction of different dopants can result in alterations to the size of the defect layer [38]. The PA-1-1 sample exhibited the lowest I_G_/I_D_ value (1.224), indicating that this ammonium dihydrogen phosphate-doped sample has a greater number of defect sites and a thicker graphitization layer, which is more conducive to electrochemical energy storage [39].

Furthermore, Figure S3a illustrates the XRD patterns of PA-1-Y, demonstrating that the diffraction peak positions of the materials remained essentially unaltered as the ratio of the KOH activator was varied. This indicates that the introduction of KOH did not significantly impact the amorphous carbon structure. The Raman spectra of PA-1-Y are presented in Figure S3b. It is notable that when no activator KOH is added, PA-1-0 has the largest I_G_/I_D_ ratio, indicating that it has the worst degree of disorder. In contrast, PA-1-1 possesses the smallest I_G_/I_D_, suggesting that when the mass ratio of KOH to peanut shell hydrocode is 1:1, the degree of defects is larger compared to the other three samples, and it can exhibit more active sites, thus exhibiting a more favorable capacity for capacitance improvement. As the KOH ratio increases (PA-1-1, PA-1-2, PA-1-3), the I_G_/I_D_ ratios gradually become larger. This indicates that an excessive KOH is unfavorable to the activation effect of peanut shell hydrocode, and thus does not result in a favorable degree of disorder. It is evident that the I_G_/I_D_ ratios of all KOH-activated samples (PA-1-1, PA-1-2, PA-1-3) are considerably smaller than those of PA-1-0. This suggests that KOH can markedly enhance the degree of defects in peanut shell-based carbon materials.

**Figure 4:**
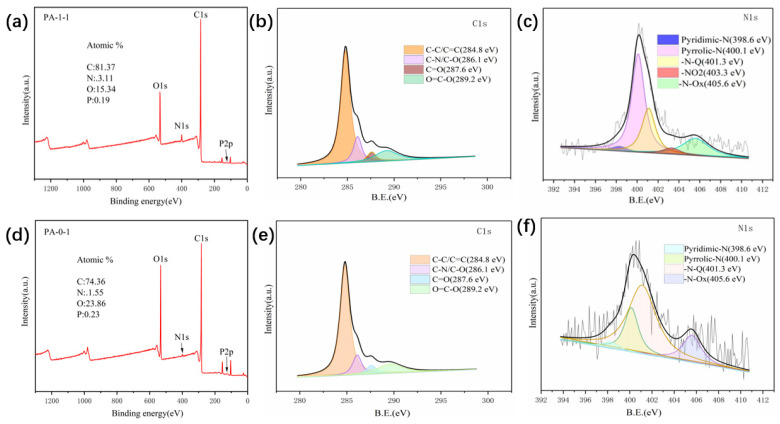
Wide-scan XPS spectrum of (**a**) PA-1-1. XPS spectrum of PA-1-1 for (**b**) C 1s and (**c**) N 1s regions. Wide-scan XPS spectrum of (**d**) PA−0−1. XPS spectrum of PA−0−1 for (**e**) C 1s and (**f**) N 1s regions.

To analyze the changes in surface elements and properties of peanut shell-based carbon materials before and after nitrogen doping, XPS characterization was conducted on samples PA-0-1 and PA-1-1. The nitrogen content of PA-0-1 increased from 1.55% (Figure 4d) to 3.11% (Figure 4a) following the addition of ammonium dihydrogen phosphate. This indicates that the introduction of nitrogen was successful. However, the P content decreased from 0.23% (Figure 4d) to 0.19% (Figure 4a) after the addition of ammonium dihydrogen phosphate, which indicates that the P element was not introduced. Figure 4b and Figure 4e present a comparative analysis of the C 1s core-level XPS spectra of the PA-0-1 and PA-1-1 samples. Four peaks at 284.8, 286.1, 287.6, and 289.2 eV are observed, corresponding to C-C/C=C, C-N/C-O, C=O, and O=C-O, respectively [40]. The N 1s XPS spectra of PA-1-1 (Figure 4c) and PA-0-1 (Figure 4f) exhibit the four peaks at 398.6, 400.1, 401.3, and 405.6 eV, which correspond to Pyridinic-N (N-6), Pyrrolic-N (N-5), Graphitic-N (G-N), and N-oxide (N-O), respectively [40,41]. As illustrated in Figure 4c,f, the content of PA-1-1 at 400.1 eV is increased following nitrogen doping. This indicates that PA-1-1 introduced more N-5. The introduction of N-5 in PA-1-1 leads to an increased number of exposed edge sites, resulting in a greater degree of defects in the carbon structure [40,42]. This phenomenon contributes to the generation of high capacitance.

**Figure 5:**
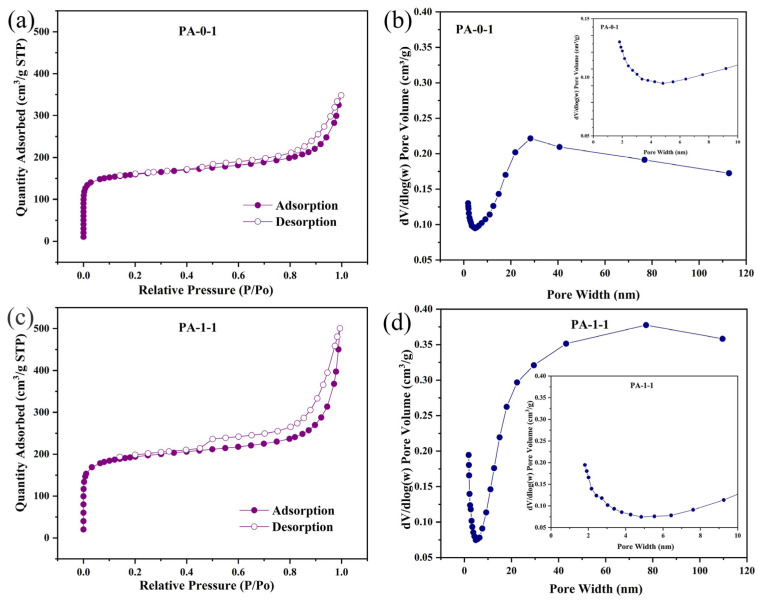
N_2_ adsorption-desorption isotherms of (**a**) PA−0−1, the pore size distribution of (**b**) PA−0−1, N_2_ adsorption-desorption isotherms of (**c**) PA-1-1 and pore size distribution of (**d**) PA-1-1.

The structure of the electrode material has a significant effect on ion transport and consequently affects the electrochemical properties of the material [43]. To investigate the structure properties of the samples, the PA-0-1 and PA-1-1 samples underwent nitrogen sorption isotherms and pore size distribution analysis. Figure 5a,b illustrate the N_2_ adsorption-desorption isotherms of the PA-1-1 and PA-0-1 samples. The isotherm curves of the two samples exhibited hybrid characteristics, classified as type I and IV according to the IUPAC classification. The H4 hysteresis loop indicated the presence of micropores and mesopores [44]. At a low relative pressure range (P/P_0_ = 0–0.05), the isotherms of the obtained PA-0-1 and PA-1-1 exhibited a steep N_2_ adsorption, indicating the presence of abundant micropores as well as a hysteresis loop, which reflects the presence of mesopores. The H4 hysteresis loop observed in the isotherm can be ascribed to the mesoporous structure and capillary condensation, occurring at a relatively higher pressure (P/P_0_ greater than 0.4) [45]. The pore size distribution plots presented in Figure 5c,d support the presence of both micropores and mesopores in the pristine (undoped) and nitrogen-doped biochar derived from peanut shells. The majority of the mesopores fall within the pore size range of 2–5 nm, which can facilitate the migration of electrolyte ions and provide ion adsorption sites.

Table S1 provides a summary of the structure properties of the PA-0-1 and PA-1-1 samples. It is evident that the specific surface area increases from 489.0 m^2^/g to 602.7 m^2^/g and the pore volume also increases from 0.31 cm^2^/g to 0.50 cm^2^/g after the addition of the nitrogen dopant. A comparable pattern is observed in the total pore volume and the average pore width of the carbonate materials.

### 2.2. Electrochemical Performance

**Figure 6:**
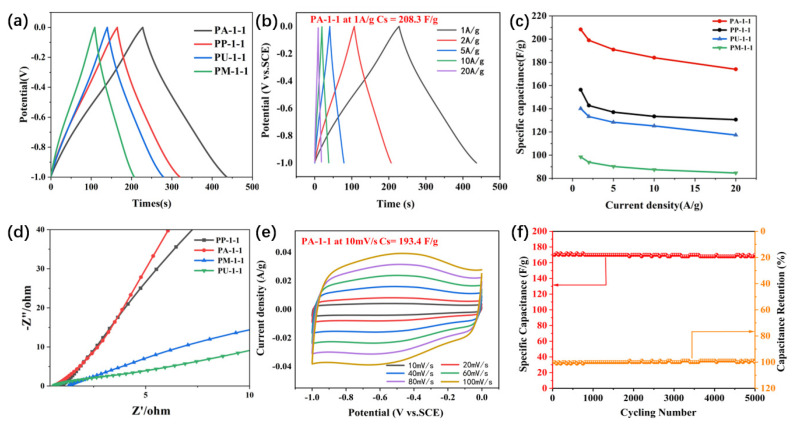
(**a**) GCD curves of all PX-1-1 (X = A, P, U, M) electrodes at a current density of 1 A/g. (**b**) GCD curves of PA-1-1 at the current densities of 1, 2, 5, 10, and 20 A/g. (**c**) Rate performance of PX-1-1 electrodes at the current density of 1, 2, 5, 10, and 20 A/g. (**d**) Nyquist plots of PX-1-1. (**e**) CV curves of PA-1-1 at various scan rates of 10–100 mV/s. (**f**) Cycling performance of PA-1-1 at a current density of 20 A/g.

The electrochemical performance of the PX-1-1 electrodes was investigated in a three-electrode system with a potential window between −1 and 0 V with 6 M KOH as the aqueous electrolyte, given its high ionic conductivity and small radius of the hydration sphere (3.00 Å for OH^−^ and 3.31 Å for K^+^) [46]. Figure 6a presents a summary of the GCD curve of the PX-1-1 electrode at a current density of 1 A/g. All the GCD curves exhibit a relatively symmetrical profile, indicative of optimal coulombic efficiency and capacitive properties [47]. In particular, the PA-1-1 electrode exhibited a longer discharge time, which can be taken to imply that it has an excellent capacitance. At a current density of 1 A/g, the calculated specific capacitances of the PM-1-1, PU-1-1, PP-1-1, and PA-1-1 electrodes are 97.9, 139.7, 155.6, and 208.3 F/g, respectively. The GCD curves of the PX-1-1 electrodes at different constant current densities, ranging from 1A/g to 20A/g, are shown in Figure 6b and Figure S4a–d. It can be observed that the GCD curves of all these samples exhibit a relatively triangular and symmetric shape. Furthermore, a minor discrepancy can be observed between the charging/discharging curve and the straight line, which can be attributed to the ohmic resistance of the electrode or the pseudo-capacitance generated by the functional groups on the surface of the electrode materials [48]. These findings suggest that all the prepared PX-1-1 electrodes exhibit satisfactory reversible charge/discharge properties. However, the incorporation of ammonium dihydrogen phosphate appears to be more effective than other nitrogen dopants in enhancing the electrochemical performance of the electrode materials. The corresponding rate performance based on the GCD data is presented in Figure 6c. Clearly, the PA-1-1 electrode delivered an excellent rate performance with a capacitance of 208.3, 199.0, 191.0, and 184.0 F/g at 1, 2, 5, and 10 A/g. Even at a current density of 20 A/g, a capacitance of 174 F/g can be achieved, which is much higher than that of PP-1-1 (130.6 F/g), PU-1-1 (117.3 F/g), and PM-1-1 (84.6 F/g).

The migration/diffusion rate of ions in materials can be determined by the shape of the cyclic voltammetry (CV) curve [49]. Figure 6e presents the CV plots of the PA-1-1 electrodes at varying scanning rates, spanning from 10 to 100 millivolts per second (mV/s). As illustrated in Figure S5, the CV curves of the PX-1-1 electrodes exhibit a rectangular shape, indicating that they exhibit a typical double-layer capacitance during the energy storage process. It can be observed that the PA-1-1 electrode exhibits a maximum specific capacitance of 193.4 F/g, which is higher than that of the PU-1-1 (131.3 F/g), PP-1-1 (140 F/g), and PM-1-1 (93.1 F/g) electrodes, respectively. This indicates that ammonium dihydrogen phosphate is a more suitable nitrogen doping agent for peanut shells than the other three nitrogen dopants. Ammonium dihydrogen phosphate may not only serve as the N resource, but also as the “activator” to expose more of the active component N, which is consistent with the findings from our previous work [50].

To understand the mechanism of energy storage, the CV data were further analyzed using Dunn’s method [51]. The contribution of capacitance and diffusion limitations to the total capacitance can be quantified further by the following equation:

i(V) = k_1_v + k_2_v^1/2^ (1)

where k_1_v represents the capacitance contribution, while k_2_v^1/2^ represents the diffusion contribution. As illustrated in Figure S6, the capacitance contribution of the PA-1-1 electrode exhibits an increase from 88.07% at 10 mV/s to 96.88% at 100 mV/s, showing a noticeable capacitive contribution.

To further provide insight into the electrochemical performance, the electrochemical impedance spectroscopy (EIS) of PX-1-1 was obtained. The resulting results are shown in Figure 6d. The Nyquist plot can be divided into two distinct frequency regions: a high-frequency region and a low-frequency region. The intercept of the Nyquist curves on the X-axis represents the intrinsic resistance (Rs) of the electrode material, which encompasses the internal resistance of the current collector, the electrode, and the contact resistance between them. The radius of the semicircle in the high-frequency region represents the charge transfer resistance (R_ct_), which is primarily influenced by the electric double-layer structure [52]. Furthermore, the oblique line at 45° within the EIS spectrum represents the Warburg impedance, which is indicative of the electrolyte ion diffusion and transmission characteristics into the electrode material itself [53]. To quantitatively evaluate all of these aspects in the impedance spectrum, the overall spectrum was fitted by the modified Randles circuit, which reproduces the straight line in the low frequency by the incorporation of a Warburg component in the circuit, and the depressed semicircle by a constant phase element (CPE) [54]. The detailed parameters are listed in Table S2. The fitted R_s_ values of PA-1-1, PP-1-1, PM-1-1, and PU-1-1 are 0.68, 1.02, 1.24, and 0.60 Ω, respectively. The larger R_s_ of PM-1-1 may be ascribed to its high porosity. The R_ct_ values of PA-1-1, PP-1-1, PM-1-1, and PU-1-1 are 0.47, 0.46, 1.23, and 0.45 Ω, respectively, indicative of the low charge transfer resistance [55,56]. In the low-frequency region, the straight line typically indicates the ion diffusion resistance of the material [57]. In comparison with PA-1-1, PP-1-1, PM-1-1, and PU-1-1, the straight line of PA-1-1 is closer to vertical, which indicates that PA-1-1 has a smaller ion diffusion resistance. Consequently, the results of the EIS analysis of these samples align with the shapes of their CV curves and the IR drops in their GCD curves. These observations indicate that the ammonium dihydrogen phosphate method is a suitable approach for synthesizing nitrogen-doped activated carbons for enhancing the electrochemical performance of the peanut shell-derived electrode.

Figure S7 shows the GCD curves of PA-1-Y prepared by the different mass ratios of the activator (KOH) to water coke. Clearly, the PA-1-0, PA-1-1, PA-1-2, and PA-1-3 electrodes delivered a capacitance of 34.9, 208.3, 150.9, and 135.5 F/g, respectively. Based on the GCD data, the related rate performance is illustrated in Figure S8. In addition, Figure S9 illustrates the CV curves of the PA-1-Y electrode, and similar results were found. As shown in Figure S9, the capacitance of 32.8, 193.4, 145.3, and 131.0 F/g was achieved for the PA-1-0, PA-1-1, PA-1-2, and PA-1-3 electrodes, respectively. Notably, the CV curves of the PA-1-1, PA-1-2, and PA-1-3 electrodes (Figure S9b–d) show a relative quasi-rectangle shape compared with the PA-1-0, which is ascribed to the introduction of the activator, which provides a sufficient activation process, thus offering capacitive behavior for charge storage. This indicates that the mass ratio of KOH to water coke has a great influence on the electrochemical performance, which is mainly ascribed to the change in the structure and surface chemistry of carbon materials.

As a consequence, the PA-1-1 electrode exhibited the best electrochemical performance among all the PX-1-1 and PA-1-Y samples in this work. The cycle stability of PA-1-1 was investigated for 5000 cycles of charging and discharging at 20 A/g and the result is shown in Figure 6f. The capacitance retention rate of PA-1-1 remains at 98.8% of the initial value after 5000 times of charging and discharging, indicating the good stability and great potential of the material in long-term cycle applications. The specific capacitances of several activated carbons prepared using other carbon precursors are listed in Table 1 to further evaluate the electrochemical performance of the PA-1-1 electrode. The as-synthesized PA-1-1 shows a relatively higher specific capacitance. In addition, the capacitance of PA-1-1 is comparable to that of carbon synthesized from some special biomass materials such as reed straw, bean sprouts, Agave angustifolia leaves, and cornstalks.

**Figure 7:**
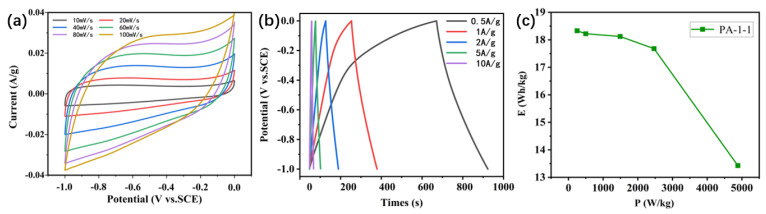
Electrochemical performance of PA-1-1 in the two-electrode system: (**a**) CV curves at different scan rates of 10–100 mV/s. (**b**) GCD curves of PA-1-1 at the current densities of 0.5, 1, 2, 5, and 10 A/g. (**c**) The Ragone plot of symmetric supercapacitors composed of PA-1-1.

To assess the practical applicability of the as-prepared biomass-based carbon electrode materials, the electrochemical performance of the PA-1-1//PA-1-1 symmetrical supercapacitor (SSC) was evaluated in a 6 M KOH electrolyte [67]. The CV curves of PA-1-1 at varying scan rates (10 mV/s to 100 mV/s) are presented in Figure 7a. It can be observed that the shape of the CV curves undergoes a gradual deformation with the increase in the scanning rate, while the CV curve still exhibits a quasi-rectangular shape with approximately mirror symmetry at a high scanning rate of 100 mV/s, indicating that the electrode exhibited ideal electrochemical capacitive behavior [68]. Figure 7b illustrates the GCD curves of PA-1-1 in a current density range from 0.5 to 10 A/g, exhibiting a triangular and symmetrical shape. This further confirms the ideal capacitive behavior of PA-1-1. Figure 7c presents the Ragone plot of PA-1-1//PA-1-1 SSC. The SSC achieved a maximum energy density of 18.33 Wh/kg at a power density of 249.57 W/kg, and an energy density of 13.43 Wh/kg at a maximum power density of 4882.83 W/kg.

## 3. Experimental Methods

### 3.1. Materials

Melamine (C_3_H_6_N_6_, AR grade), ammonium dihydrogen phosphate (H_6_NO_4_P, AR grade), urea (CO(NH_2_)_2_, AR grade), urea phosphate (CO(NH_2_)_2_-H_3_PO_4_, AR grade), potassium hydroxide (KOH, AR grade) were purchased from Aladdin Reagent (Shanghai, CN) Co. Acetylene black (AB), polyvinylidene fluoride (PVDF), concentrated hydrochloric acid (HCl), and N-methyl pyrrolidone (NMP) were purchased from Chengdu Kelong Chemical Reagent Company (Chengdu, CN). N_2_ (high purity) was purchased from the Shuangliu County Guangdu Gas Operation Department (Chengdu, CN). All the chemicals were used without further purification. Peanut shell powder was bought from the Lianfeng Agricultural Products Deep Processing Taobao store (Lianyungang, CN).

### 3.2. Preparation of Electrode Material

#### 3.2.1. Preparation of Biochar

A 100 mL beaker was filled with 3 g of peanut shell powder, followed by the addition of 3 g of nitrogen dopant (melamine, urea, ammonium dihydrogen phosphate, or uranyl phosphate) and 60 mL of deionized water. The mixture was stirred continuously until the peanut shell powder had dispersed in the water. The mixture was then loaded into a 100 mL hydrothermal kettle and sealed. The hydrothermal kettle was heated to a constant temperature of 200 °C for 8 h in a drying oven. After cooling, the yellowish-brown mixture was rinsed with deionized water until the effluent turned colorless. The washed samples were then dried at 80 °C for 12 h to obtain yellow-brown aqueous coke powder, which was labeled PX (X denotes different nitrogen dopants, where M = melamine, U = urea, A = ammonium dihydrogen phosphate, and P = uranyl phosphate).

#### 3.2.2. Activation Process

A 100-milliliter beaker should be prepared, and then 1 g of PX powder and 1 g of KOH powder should be added. Subsequently, 10 mL of deionized water should be added, the beaker should be sealed with cling film, and the mixture should be stirred continuously for four hours. The mixture should be dried in a drying chamber at 80 °C for 12 h to obtain a dark brown aqueous char and KOH powder. The mixture was subjected to activation in a tube furnace at 600 °C for two hours, from 20 °C to 600 °C, at a rate of 5 °C per minute, under a high-purity N_2_ flow. Subsequently, the activation products were washed with 1 M HCl and then distilled. Subsequently, the samples were dried in a vacuum oven at 60 °C for six hours. Subsequently, the activated carbon material was pulverized to a fine powder, packaged in bags, and labeled as PX-1-1, where X stands for different nitrogen dopants (M for melamine, U for urea, A for ammonium dihydrogen phosphate, and P for uranyl phosphate); the first 1 corresponded to the mass ratio of peanut shell hydrocode to peanut shell, and the second 1 represented the mass ratio of KOH to peanut shell hydrocode.

### 3.3. Materials Characterizations

The crystal structure was analyzed using an X-ray diffractometer (XRD, DX-2700BH, Haoyuan Instrument Co., Ltd., Dandong, CN) with Cu Kα radiation, operated at 40 kV and 30 mA, in the range of 10–70°. Raman spectra were recorded using a DXR Raman Microscope (Thermo Fisher Scientific, Inc., Waltham, MA, USA). The surface area was calculated by the Brunauer–Emmett–Teller method (BET), utilizing the ASAP 2460 (Micromeritics Instrument, CORP., Norcross, GA, USA) accelerated surface area and porosimetry system, employing N_2_ gas at −196 °C. The surface morphologies were analyzed using FE-SEM (Field Emission Scanning Electron Microscope) REGULUS 8230 (Hitachi, Co., Ltd., Tokyo, Japan). The surface binding states and elemental composition were characterized by X-ray photoelectron spectroscopy (XPS), utilizing a Kratos Analytical, Ltd. (Manchester, UK) AXIS ULTRA DLD system.

### 3.4. Electrochemical Measurement

The collector was made from nickel foam. It was cut into pieces and cleaned with 1 M HCl and water. It was then dried at 60 °C. Meanwhile, a binder solution was made with polyvinylidene fluoride and azomethylpyrrolidone. The active material solution (ink) was made by mixing polyvinylidene fluoride (10 wt.%), acetylene black (10 wt.%), and activated carbon (80 wt.%), adding azomethylpyrrolidone and mixing. The ink was carefully dropped on a nickel foil collector (1 cm × 1 cm) and dried at 60 °C for 8 h. A tablet press machine was used to press dried collectors at 10 MPa, and the electrode with a mass loading of approximately 1 mg was obtained. Finally, the collector was used as a working electrode in a three-electrode system with a saturated calomel electrode, a platinum sheet, and a KOH electrolyte.

Cyclic voltammetry and constant current charge/discharge were performed on a CHI600E electrochemical workstation from Shanghai Chenhua Instrument Co. in China. The current density of the GCD was 1–20 A/g. The scan rate of the CV was 10–100 mV/s. The capacitance was calculated from the GCD curves.

C = I × Δt/(m × ΔV) (2)

ΔV (V) is the potential window, m (g) is the mass loading of the active substance, Δt (s) is the discharge time, I (A) is the current density, and C (F/g) is the specific capacitance. The energy density (E, Wh/kg) and the power density (P, W/kg) were obtained by the galvanostatic discharge tests according to the following equations:

E = 1/2C × ΔV^2^/3.6 (3)

P = E × Δt × 3600(4)

## 4. Conclusions

In conclusion, nitrogen-doped porous carbons have been successfully synthesized from peanut shells via a three-step process: hydrothermal carbonization, KOH activation, and nitrogen doping. The type of nitrogen dopants and the amount of the activating agent (KOH) were investigated. The rational amount of KOH and the nitrogen resources were found to be significant factors influencing the electrochemical performance of the peanut shell-based electrode. The optimized PA-1-1 was prepared with ammonium dihydrogen phosphate as the nitrogen dopant, with a mass ratio of KOH to peanut shell hydrocode of 1:1. It possessed a N content of 3.11% and a specific surface area of 602.7 m^2^/g. In 6 M KOH, the PA-1-1 electrode displayed a specific capacitance of 208.3 F/g and reached 170.0 F/g at 20 A/g with a capacitance retention of 81.6%. In addition, the PA-1-1 electrode exhibited excellent cycling stability with a capacitance retention of 98.8% after 5000 cycles. The PA-1-1//PA-1-1 symmetric supercapacitor exhibited an energy density of 17.7 Wh/kg at a power density of 2467.0 W/kg. The nitrogen-doped peanut shell-based porous carbon is believed to be a highly promising material for energy storage applications.

## Figures and Tables

**Table 1:** Comparison of the capacitance of PA-1-1 with other biomass-derived activated carbons reported.

Carbon Precursor	Activating Agent	Electrolyte Used	Highest Specific Capacitance (F/g)	Ref.
Peanut shells	KOH	6 M KOH	208.3	This work
Reed straw	KOH	6 M KOH	202.8	[46]
Casein	C_6_H_5_K_3_O_7_	3 M KOH	177	[58]
Bean sprouts	/	1 M KOH	203.8	[59]
Koiralo flower	KOH	6M KOH	165	[60]
Durian shells	(NH4)_2_HPO_4_	1 M H_2_SO_4_	184	[61]
Agave angustifolia leaves	K_2_CO_3_	1 M Na_2_SO_4_	200	[62]
Tea	KOH	1 M KOH	167	[63]
Cornstalk	K_2_CO_3_	6 M KOH	203.5	[64]
Durian husk	NaOH	1 M TEABF_4_.	145	[65]
Paper flower	ZnCl_2_	1 M H_2_SO_4_	118	[66]

## Data Availability

Data are contained within the article.

## Supplementary Materials

The following supporting information can be downloaded at: https://www.mdpi.com/article/10.3390/ijms25147583/s1.
