# Supplementary material for: Nitrogen-Doped Porous Carbons Derived from Peanut Shells as Efficient Electrodes for High-Performance Supercapacitors
Source: Int J Mol Sci. 2024 Jul 10;25(14):7583. doi: 10.3390/ijms25147583 (PMC11277184; doi:10.3390/ijms25147583)
Supplement: Supplementary file 1 [file ijms-25-07583-s001.zip › ijms-3060296-supplementary.pdf]

## Supplementary Information

# Nitrogen-Doped Porous Carbons Derived from Peanut Shells as Efficient Electrodes for High-Performance Supercapacitors

**Shibo Liu, Qishan Zhang, Jiani Liu, Jiarui Li, Wenjia Liu, Yuan Wang * and Shaojun Yuan ***

Low-Carbon Technology & Chemical Reaction Engineering Lab, College of Chemical Engineering, Sichuan University, Chengdu 610065, China; liushibo@stu.scu.edu.cn (S.L.); zhangqishan@stu.scu.edu.cn (Q.Z.); liujiani@stu.scu.edu.cn (J.L.); 2022141490313@stu.scu.edu.cn (J.L.); 19980811423@163.com (W.L.)

* Correspondence: wangyuan2022@scu.edu.cn (Y.W.); ysj@scu.edu.cn (S.Y.); Tel./Fax: +86-28-85405201 (S.Y.)

## Supplementary Results

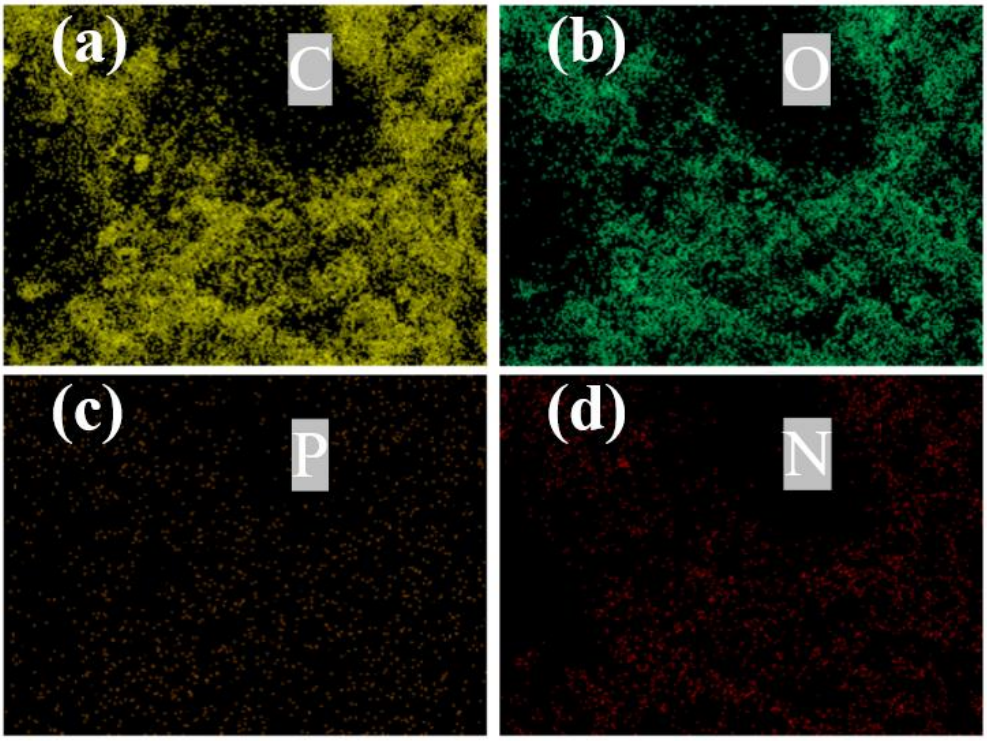


**Figure S1.** EDS mapping images of peanut shell-based material PA-1-1.

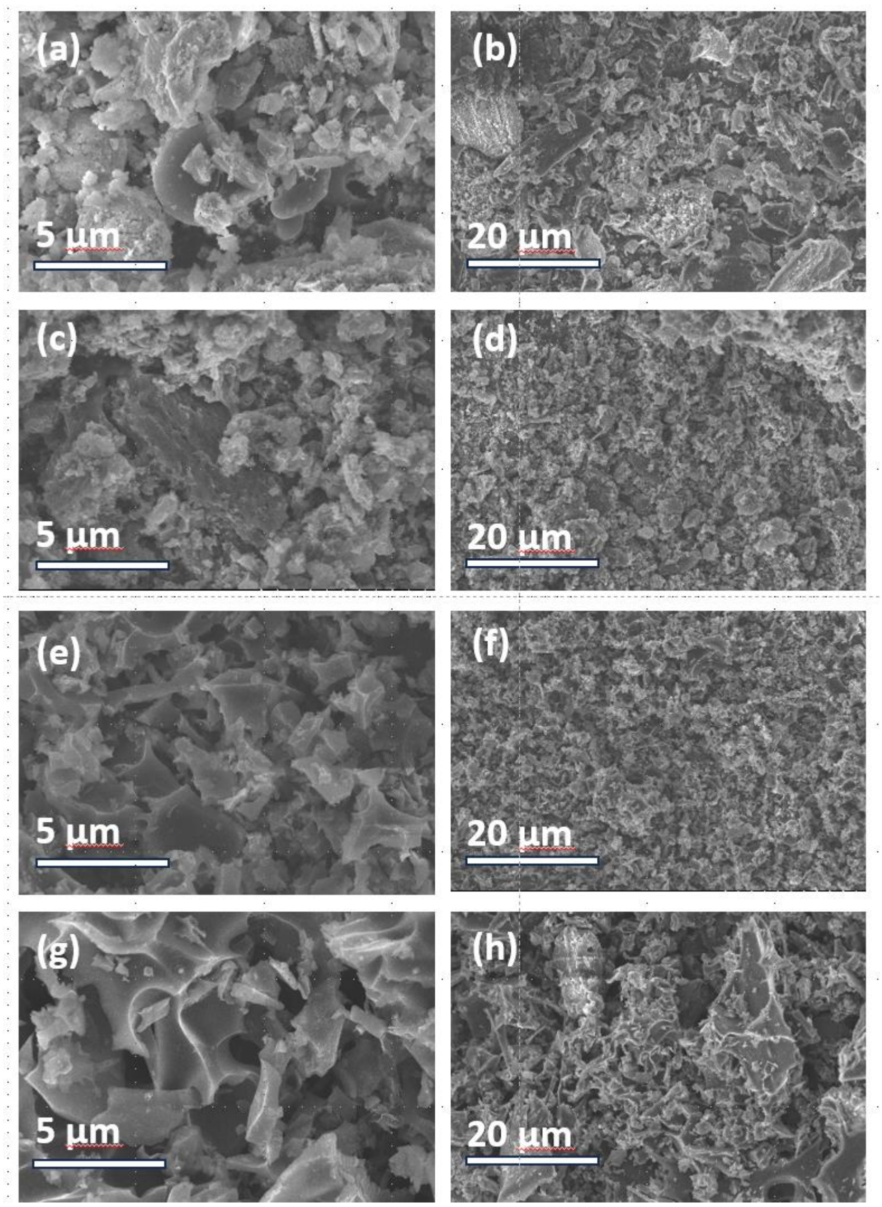


**Figure S2.** SEM of peanut shell-based materials PA-1-Y: (a, b) PA-1-0, (c, d) PA-1-1, (e, f) PA-1-2, and (g, h) PA-1-3.

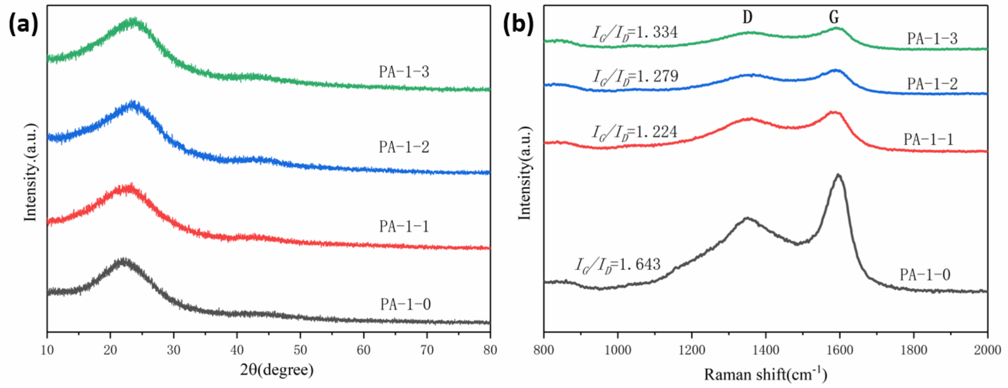


**Figure S3.** (a) XRD patterns of PA-1-Y and (b) Raman spectra of PA-1-Y.

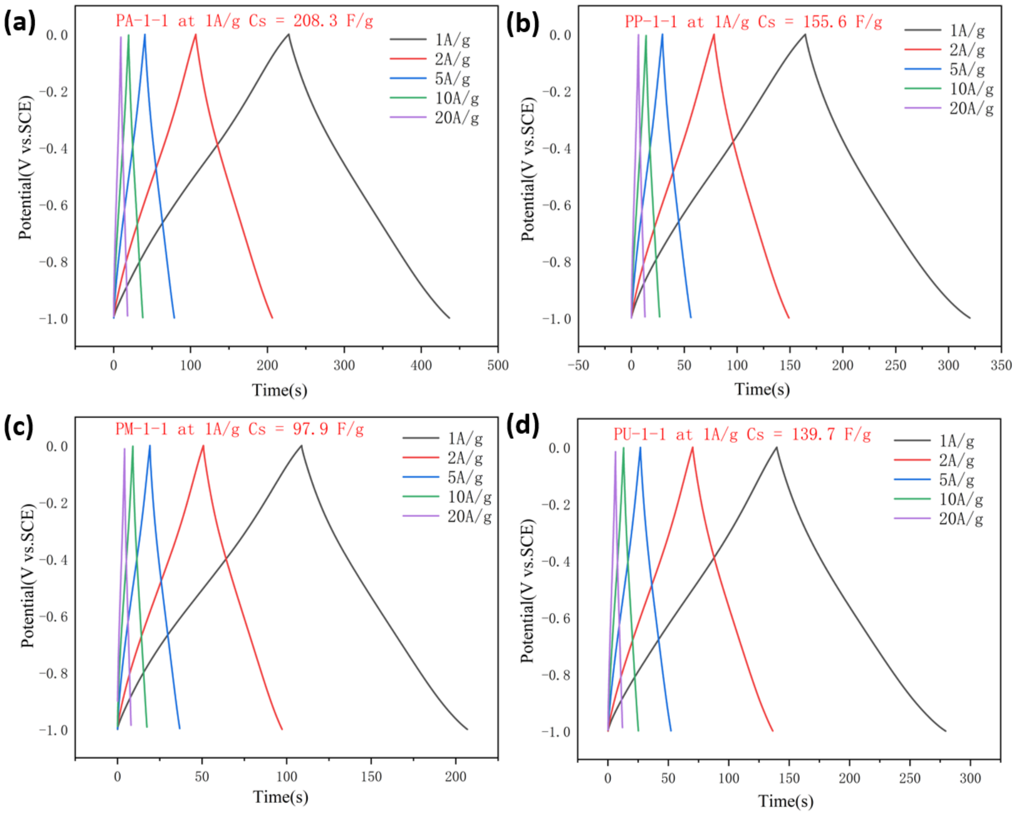


**Figure S4.** GCD curves of (a) PA-1-1, (b) PP-1-1, (c) PM-1-1, and (d) PU-1-1 at different current densities of 1, 2, 5,10, and 20 A/g.

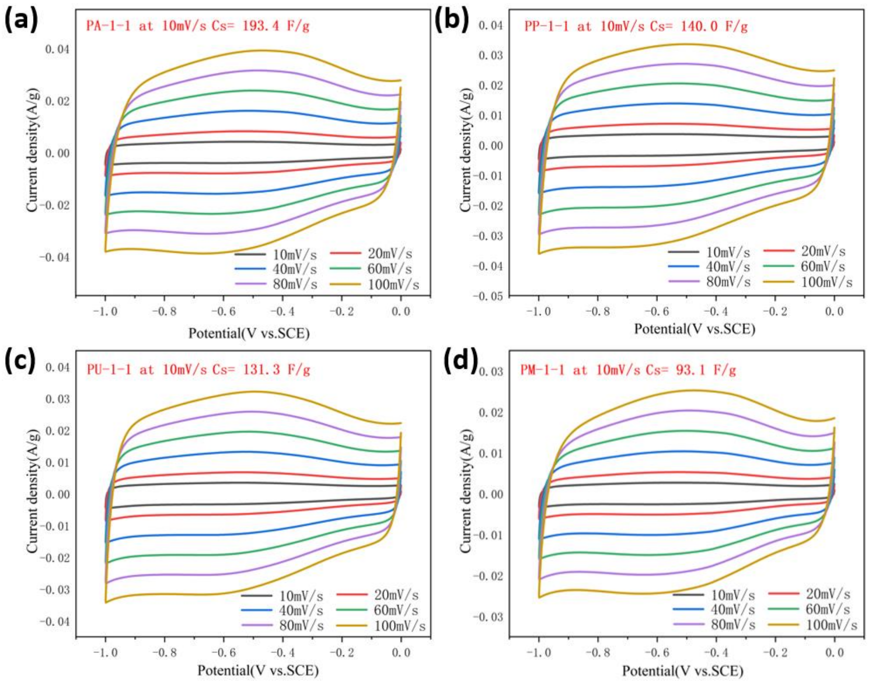


**Figure S5.** CV curves of (a) PA-1-1, (b) PP-1-1, (c) PU-1-1, and (d) PM-1-1 electrodes at a scan rate of 10–100 mV/s.

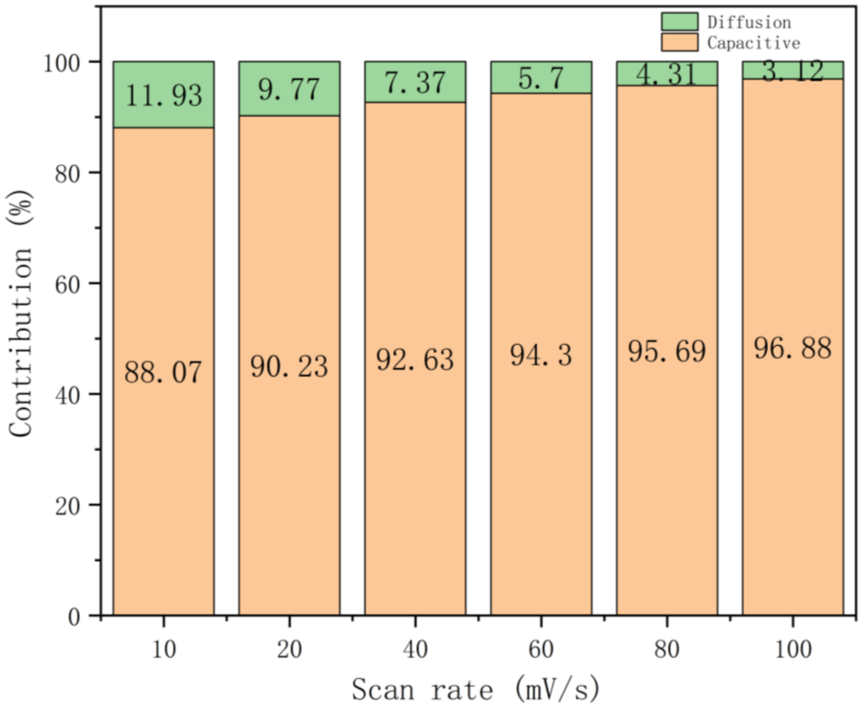


**Figure S6.** Kinetic behavior of PA-1-1 at different scan rates.

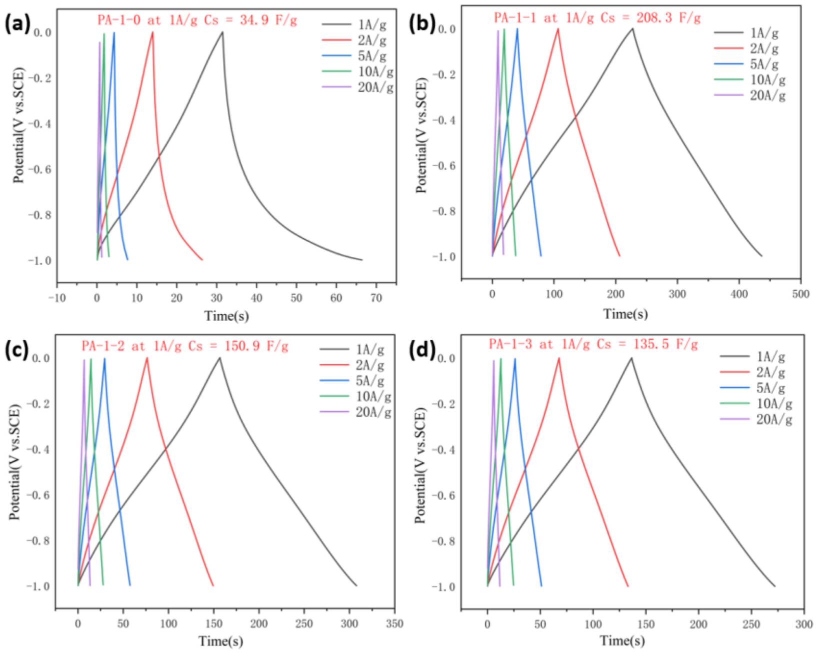


**Figure S7.** GCD curves of (a) PA-1-0, (b) PA-1-1, (c) PA-1-2, and (d) PA-1-3 electrodes at different current densities of 1, 2, 5,10, and 20 A /g.

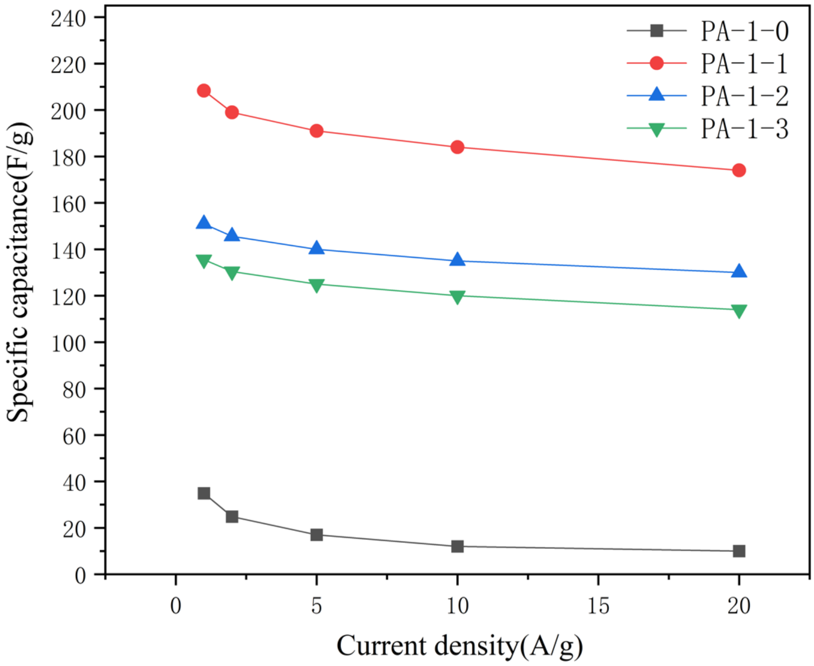


**Figure S8.** Rate performance of PA-1-Y at the current density of 1, 2, 5, 10, and 20 A/g.

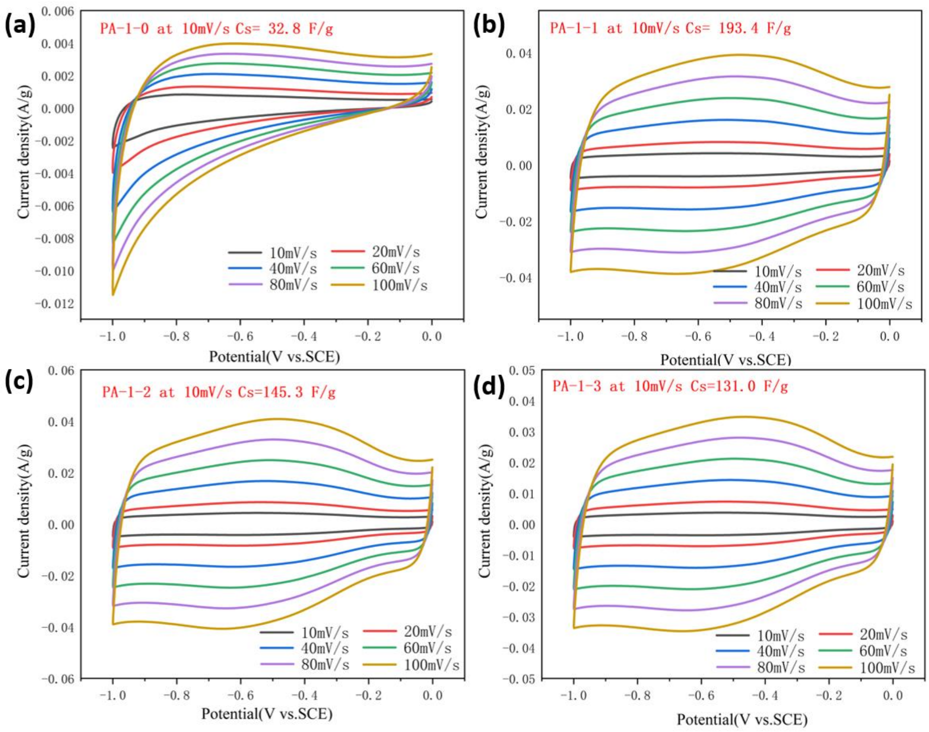


**Figure S9.** CV curves of (a) PA-1-0, (b) PA-1-1, (c) PA-1-2, and (d) PA-1-3 electrodes at a scan rate of 10–100 mV/s.

**Table S1.** Textural properties data of the pristine and nitrogen-doped samples

| Sample | Adsorption/ Desorption | BET Surface Area ($m^2/g$) | Average Pore Width (nm) | Pore Volume ($cm^2/g$) |
|---|---|---|---|---|
| PA-0-1 (undoped) | A | 488.9726 | 10.4558 | 0.303173 |
| | D | | 10.6073 | 0.314254 |
| PA-1-1(doped) | A | 602.7140 | 13.1223 | 0.453513 |
| | D | | 12.4247 | 0.501666 |

**Table S2.** Parameters in the modified Randles circuit

| | Rs | Rct | Wo-R | Wo-P | CPE-P |
|---|---|---|---|---|---|
| PA-1-1 | 0.68 | 0.47 | 3.48 | 0.42 | 1.10 |
| PP-1-1 | 1.02 | 0.46 | 5.74 | 0.41 | 1.07 |
| PU-1-1 | 0.60 | 0.45 | 9.77 | 0.58 | 0.88 |
| PM-1-1 | 1.24 | 1.23 | 4.58 | 0.65 | 0.72 |
